# Cortex-restricted deletion of *Foxp1* impairs barrel formation and induces aberrant tactile responses in a mouse model of autism

**DOI:** 10.1186/s13229-023-00567-0

**Published:** 2023-09-11

**Authors:** Xue Li, Shishuai Hao, Shimin Zou, Xiaomeng Tu, Weixi Kong, Tian Jiang, Jie-Guang Chen

**Affiliations:** 1 State Key Laboratory of Ophthalmology, Optometry and Visual Science, Eye Hospital, Wenzhou Medical University, 270 Xueyuan Road, Wenzhou, 325027 Zhejiang People’s Republic of China; 2 School of Biomedical Engineering, Wenzhou Medical University, Wenzhou, 325027 People’s Republic of China; 3 Research Center for Translational Medicine, The Affiliated Wenling Hospital of Wenzhou Medical University, Wenling, 317500 People’s Republic of China

**Keywords:** Autism, Tactile, Barrel cortex, Thalamocortical, c-Fos, Spines

## Abstract

**Background:**

Many children and young people with autism spectrum disorder (ASD) display touch defensiveness or avoidance (hypersensitivity), or engage in sensory seeking by touching people or objects (hyposensitivity). Abnormal sensory responses have also been noticed in mice lacking ASD-associated genes. Tactile sensory information is normally processed by the somatosensory system that travels along the thalamus to the primary somatosensory cortex. The neurobiology behind tactile sensory abnormalities, however, is not fully understood.

**Methods:**

We employed cortex-specific *Foxp1* knockout (*Foxp1*-cKO) mice as a model of autism in this study. Tactile sensory deficits were measured by the adhesive removal test. The mice’s behavior and neural activity were further evaluated by the whisker nuisance test and c-Fos immunofluorescence, respectively. We also studied the dendritic spines and barrel formation in the primary somatosensory cortex by Golgi staining and immunofluorescence.

**Results:**

*Foxp1*-cKO mice had a deferred response to the tactile environment. However, the mice exhibited avoidance behavior and hyper-reaction following repeated whisker stimulation, similar to a fight-or-flight response. In contrast to the wild-type, c-Fos was activated in the basolateral amygdala but not in layer IV of the primary somatosensory cortex of the cKO mice. Moreover, *Foxp1* deficiency in cortical neurons altered the dendrite development, reduced the number of dendritic spines, and disrupted barrel formation in the somatosensory cortex, suggesting impaired somatosensory processing may underlie the aberrant tactile responses.

**Limitations:**

It is still unclear how the defective thalamocortical connection gives rise to the hyper-reactive response. Future experiments with electrophysiological recording are needed to analyze the role of thalamo-cortical-amygdala circuits in the disinhibiting amygdala and enhanced fearful responses in the mouse model of autism.

**Conclusions:**

*Foxp1*-cKO mice have tactile sensory deficits while exhibit hyper-reactivity, which may represent fearful and emotional responses controlled by the amygdala. This study presents anatomical evidence for reduced thalamocortical connectivity in a genetic mouse model of ASD and demonstrates that the cerebral cortex can be the origin of atypical sensory behaviors.

## Background

Autism spectrum disorder (ASD) is a complex neurodevelopmental disorder characterized by deficiencies in language acquisition, difficulties in social interactions, and stereotyped interests [1]. Many children diagnosed with autism tend to process sensory information differently than individuals without autism, resulting in hyper- or hypo-reactivity to external sensory stimuli, or a combination of both [2, 3]. Atypical sensory processing is a crucial feature of ASD and has become a diagnostic criterion, as reported in the Diagnostic and Statistical Manual of Mental Disorders (DSM-V, 2013). Among multiple sensory modalities (taste, touch, audition, smell, and vision) [4], tactile processing dysfunction is one of the most frequent findings in patients with ASD [5, 6]. Many children and young people with ASD may display touch defensiveness or avoidance (hypersensitivity) or engage in sensory seeking by touching people or objects (hyposensitivity) [7].

The somatosensory system in mammals relays sensations detected in the periphery and transmits them through the spinal cord, brainstem, and thalamocortical projection to the sensory cortex in the brain. Facial vibrissae, commonly known as whiskers, are one of the most developed tactile perception organs in rodents. Tactile information from the whiskers is relayed through thalamic nuclei to layer IV of the primary somatosensory cortex (S1) via excitatory spiny stellate neurons and inhibitory basket cells, the latter of which produces disynaptic feedforward inhibition in the cortical cells [8]. Neurons in layer IV of S1 are tangentially organized where each whisker on the snout is represented in a discrete and periodic anatomical unit called “barrel” [9]. During the early development of the barrel map, each bundle of thalamocortical axons (TCA), transmitting sensory information from a single principal whisker, clusters in the barrel center. Shortly after the arrival of TCA in layer IV, postsynaptic spiny stellate neurons orient their dendrites toward the barrel center and encircle the incoming TCA arborizations, forming a cell-dense barrel wall. Each barrel is separated by cell-sparse septal regions [10, 11]. Conceivably, dysfunction in the thalamocortical connectivity may result in tactile sensory deficits.

The tactile sensory disturbances have been investigated in genetic mouse models of ASD. Fragile X syndrome (FXS) is the leading single-gene (*Fmr1*) cause of autism [12]. Mice with *Fmr1* knockout (KO) overreacted to repetitive whisker stimulations, resembling tactile defensiveness in FXS patients [13, 14]. Engraield-2 (*EN2*) genetic variants are also associated with ASD [15, 16]. Mice with *En2*^*tm1Alj*^ targeted mutation exhibited ASD-like behaviors [17, 18], and showed sensory hyper-responsiveness in the whisker-specific behavioral test [19]. The hyper-reactive behaviors in *Fmr1* KO or *En2* mutated mice were accompanied by functional hypoconnectivity in sensory brain areas, as assessed by functional MRI imaging [19, 20]. In contrast, tactile hyposensitivity was noticed in autistic patients with SYNGAP1 haploinsufficiency. Heterozygous KO of *Syngap1* leads to reduced touch-related neural activity in the mouse S1 [21]. Despite these studies, however, the neurobiological basis beneath abnormal sensory responses, particularly hyper-reactivity, remains elusive.

Hyper-reactivity and sensory seeking are common in individuals with *FOXP1* syndrome, a neurodevelopmental disorder manifesting characteristic symptoms of ASD [22–25]. *FOXP1* gene belongs to the Forkhead Box P (FOXP) subfamily of transcription factors that regulates the development of multiple organs [26, 27]. Within the mouse brain, *Foxp1* is expressed in the cortex (layer III-VI), striatum, and CA1/CA2 region of the hippocampus [28], and is a key regulator of neural development [29, 30]. Conventional *Foxp1*^+/-^ mice displayed deficits in ultrasonic vocalization production [31]. Conditional KO of *Foxp1* in the brain leads to striatum developmental defects and autism-like behaviors in mice [32]. Moreover, loss of *Foxp1*, specifically in the pyramidal neurons of the neocortex and hippocampus, leads to intellectual disability, increased anxiety, communication impairments, and decreased sociability [29], indicating that the cerebral cortex may be the origin of ASD-like behaviors.

To investigate neuronal mechanisms underlying the tactile sensory abnormality related to ASD, we explored the aforementioned cortex-specific *Foxp1* knockout (*Foxp1*-cKO) mice in this study. We found that *Foxp1*-cKO mice had delayed tactile sensation while exhibited hyper-reactivity to repeated whisker stimulation. *Foxp1* deficiency in excitatory cortical neurons disrupted barrel formation, decreased dendritic spines, reduced c-Fos immunochemistry in layer IV of S1, and increased c-Fos in the basolateral amygdala. These findings suggest that *Foxp1* plays an essential role in thalamocortical connectivity, the loss of which may lead to atypical sensory responses.

## Methods

### Animals

*Foxp1*^*flox/flox*^ mice (Jackson Laboratory, stock #017699) in a C57BL/6 J background possess *loxP* sites on either side of exons 11 and 12 of the targeted *Foxp1* gene. *Foxp1*^*flox/flox*^ mice were crossed with the *Emx1-Cre* mice, where Cre recombinase is expressed specifically in the pyramidal neurons of neocortex and hippocampus. Male *Foxp1*^*flox/flox*^; *Emx1-Cre*^+^ progenies were crossed with female *Foxp1*^*flox/flox*^ mice to generate homozygous, forebrain-specific *Foxp1* knockout mice. DNA was extracted from the tip of the tail, and the mice were genotyped using standard polymerase china reaction (PCR) with the following primers: for the *Foxp1*^*flox/flox*^ F-5′-TGGTTCACACGAATGTTTGC-3′ and R-5′-GGAGTGGCTCTTCCATCTGA-3′ to detect mutant (300-bp product) and wild-type (211-bp product) alleles. *Cre* was detected using primer F-5’-TGTCACCTCCAATGACTAGGGGAAC-3′ and R-5′- TCCAGGTATGCTCAGAAAACGCC-3′. All animal experimental procedures and husbandry were conducted in accordance with the animal handling guidelines and protocol approved by the Animal Care Committee of Wenzhou Medical University. The mice were housed in a temperature- and light-controlled environment with a standard 12-h/12-h light–dark cycle, and given ad libitum access to water and chow. The day of birth was designated postnatal day 0 (P0). We chose male mice from the litter that gave birth to 6 ~ 8 pups in our experiments.

### Behavioral experiments

Mice at 6–8 weeks of age were subjected to behavioral assays. A few days before the experiments, mice were removed from the housing room and placed in the experimental room for 1–2 h daily to acclimatize to the behavioral testing areas. To reduce the stress and anxiety of the mice, the experimenter was present with the mice in the testing room at least 10 min before the testing. Behavioral experiments were conducted at the same time of the day. All testing apparatuses were cleaned with 70% ethanol and water between trials to remove olfactory cues. The investigators were blind to the genotype during all the behavioral testing.

### Marble burying assay

For the marble burying test, a 5 cm thick corncob bedding was applied to the bottom of a novel home cage (40W × 23D × 20H cm). On top of the bedding, set 20 colored glass marbles (14 mm diameter) that were evenly spaced (four rows of five marbles per row). Mice were placed into one corner of the cage. After 30 min, the number of marbles buried by the mouse was manually evaluated. A marble was considered buried when more than two-thirds of the marble was covered by bedding.

### Adhesive removal test

An adhesive removal test was used to assess the vibrissae sensation [33]. Mice were acclimated to the testing room in their home cages for 1 h prior to testing. One experimenter held the mouse for another to place the tape on the whisker. Small adhesive tape (2 × 2 mm) was gently applied on one side of the C2 or C3 vibrissae. Make sure to keep equal pressure between each trial and animal. The order of placement (left or right) of adhesive paper was alternated at each trial. The mouse was then placed in its home cage and observed for 60 s. Four trials were performed for each mouse (at least 10 min of rest between each trial), two for the tapes on the left and two on the right side of the nose. The time to contact is defined as the time when the mouse returned to the cage to when the mouse first raised his forepaws trying to swipe off the adhesive. From raising forepaws to removing the adhesive tape is the time to remove [33]. The average score of four trials was calculated for each animal.

### Whisker nuisance test and c-Fos immunostaining

WN test was applied to detect tactile perception by manually stimulating whiskers with a wooden stick [19, 34]. Before the testing, mice were placed into a novel empty cage (experimental cage) for 30 min per day for two days to help them familiarize the environment. On the testing day, mice were allowed to explore the experimental cage freely for 30 min before the testing (pre-test). The testing phase consists of four consecutive sessions (5 min each, with 1 min intervals). During the first (sham-stimulation) session, a wooden stick (length: 20 cm; diameter: 3 mm) was placed near the mouse, but without contact with the mouse’s whiskers or body. In the following three stimulation sessions, the mice’s bilateral whiskers were manually bent by rhythmic movements of the wooden stick. Five different behavior responses were quantitatively measured: freezing, guarding, evasion behaviors, climbing, and startle events [35]. Freezing was considered when the mouse was completely immobile in a feared posture (cowered). A guarding mouse is in an active defensive state. Evasion was scored when the mouse tried to run away to avoid repeated stimulation. The time spent in freezing, guarding, and evasion behaviors were recorded. Climbing was when the mouse displayed curiosity by active exploration of the stick with the forelimbs. Startle events were counted when the mouse exhibited abrupt and uncoordinated avoidance movement. Mouse brains were collected two hours after the sham and WN test for the immunofluorescence study of c-Fos.

### Immunohistochemistry

Mice were anesthetized by intraperitoneal injection of pentobarbital (100 μg/g of body weight), and perfused transcardially with 4% paraformaldehyde (PFA) in 0.1 M sodium phosphate buffer (PBS). Brains were dissected and post-fixed with ice-cold 4% PFA for 6 h, cryoprotected in 30% sucrose in PBS, then frozen in OCT compound (Thermo, 6502). Brains were sectioned either at 40 μm for free-floating or at 14 μm for slide-mounted immunofluorescence by a cryostat (HM505E, Microm, Germany). Heat-mediated antigen retrieval was performed by incubating sections in citrate buffer (10 mM citrate, pH 6, 0.05% Tween-20) at 100 °C for 5 min. After PBS wash, brain sections were blocked with PBS containing 0.3% Triton X-100, 1% BSA, and 5% donkey normal serum, and incubated with the primary antibodies overnight at 4 °C. Following the PBS wash, the sections were incubated with secondary antibodies at room temperature for 2 h. After further rinsing, the brain sections were mounted in an anti-fade mounting medium (Invitrogen, S36938) that contains DAPI for nuclear staining. Images were taken with a confocal laser-scanning microscope (LSM880, Zeiss). The primary antibodies used were as follows: Rabbit anti-FOXP1 (1:250, Abcam, ab227649); Mouse anti-FOXP1 (1:250, Santa Cruz, sc-398811); Guinea pig anti-c-Fos (1:1000, synaptic systems, 220604); Mouse anti-VGluT2 (1:700, Abcam, ab227649); Rabbit anti-SATB2 (1:800, Abcam, ab92446); Rabbit anti-5-HT (1:500, ImmunoStar, 24330); Rabbit anti-PSD-95 (1:600, Invitrogen, 516900). All secondary antibodies were from Jackson ImmunoResearch Laboratories: Alexa Fluor^TM^488-conjugated anti-Mouse and anti-Rabbit IgGs (1:500); Cy3-conjugated anti-Rabbit (1:500); Alexa Fluor^TM^594-conjugated anti-Mouse and anti-Rabbit IgG (1:500); Alexa Fluor^TM^594-conjugated anti-Guinea pig (1:500).

### Golgi staining

Whole brains were subjected to Golgi staining using FD Rapid Golgistain™ Kit (FD Neurotechnologies, PK401) according to the specifications. Briefly, fresh brains were dissected quickly and washed with chilled Milli-Q water. Then the brain samples were immersed in 5 ml of Solutions A and B (1:1) in a 15 ml conical tube. After 24 h, the brains were transferred to a new tube with 5 ml of Solutions A and B and kept in the dark at room temperature for 13 more days. Brain tissues were transferred into Solution C and stored in the dark at 4 °C for at least 4 days. Brains were rapidly frozen in pre-cold isopentane on dry ice for 1 min and stored at − 80 °C. The brain was sectioned in coronal planes (150 μm thickness) using a cryostat (HM505E, Microm, Germany) at − 26 °C. Sections were mounted onto gelatin-coated slides (FD Neurotechnologies, PO102) using Solution C, dried naturally overnight at room temperature in the dark, then submerged in a mixture of Solutions D and E (Solution D: Solution E: Milli-Q water = 1:1:2) for 10 min. The slides were dehydrated through ascending grades of ethanol (50%, 75%, 95%, and 100%). Finally, the slides were cleared with xylene and coverslipped with a Permount mounting medium.

For dendritic analysis, layer IV neurons were reconstructed and analyzed by Neurolucida Explore software (MBF Bioscience). Dendritic spines of layer IV neurons from the primary somatosensory cortex were imaged on a Zeiss brightfield confocal microscope. Digital zoom was set at 1.6 × under 63 × magnification. A line was drawn between the end of each dendrite and the center of the soma to analyze the asymmetry of dendrite distribution. Neurons were scored as asymmetric when 50% or more of their dendrites were directed toward one quadrant [36]. A Sholl analysis was adopted to evaluate dendritic branching complexity [37]. The maximum intersections of dendritic arbors with a series of concentric circles (radii 10–140 μm from the center of soma) were counted. Dendritic spine density was determined by counting the spines along the dendrite (20 μm in length).

### Statistics analysis

Data from at least three independent experiments were used for quantification analysis. Results were reported as mean ± SD. C-Fos positive cells in the cerebral cortex, hippocampus, and BLA were counted from the confocal images of at least three brain sections for each animal. The fluorescence intensities of VGluT2 and 5-HT in layer IV were calculated by ImageJ and expressed as percentages of the WT control, assuming the control to be 100%. To compare the two groups, an unpaired Student's *t-*test was performed using GraphPad Prism 8. Comparison among multiple groups was analyzed by one-way ANOVA, followed by Tukey's post hoc multiple comparison test. *P* < 0.05 was considered as statistical significance. Asterisks indicate *p* values with *p < 0.05, **p < 0.01, and ***p < 0.001.

## Results

### *Foxp1*-cKO mice have tactile sensory deficits

**Fig. 1:**
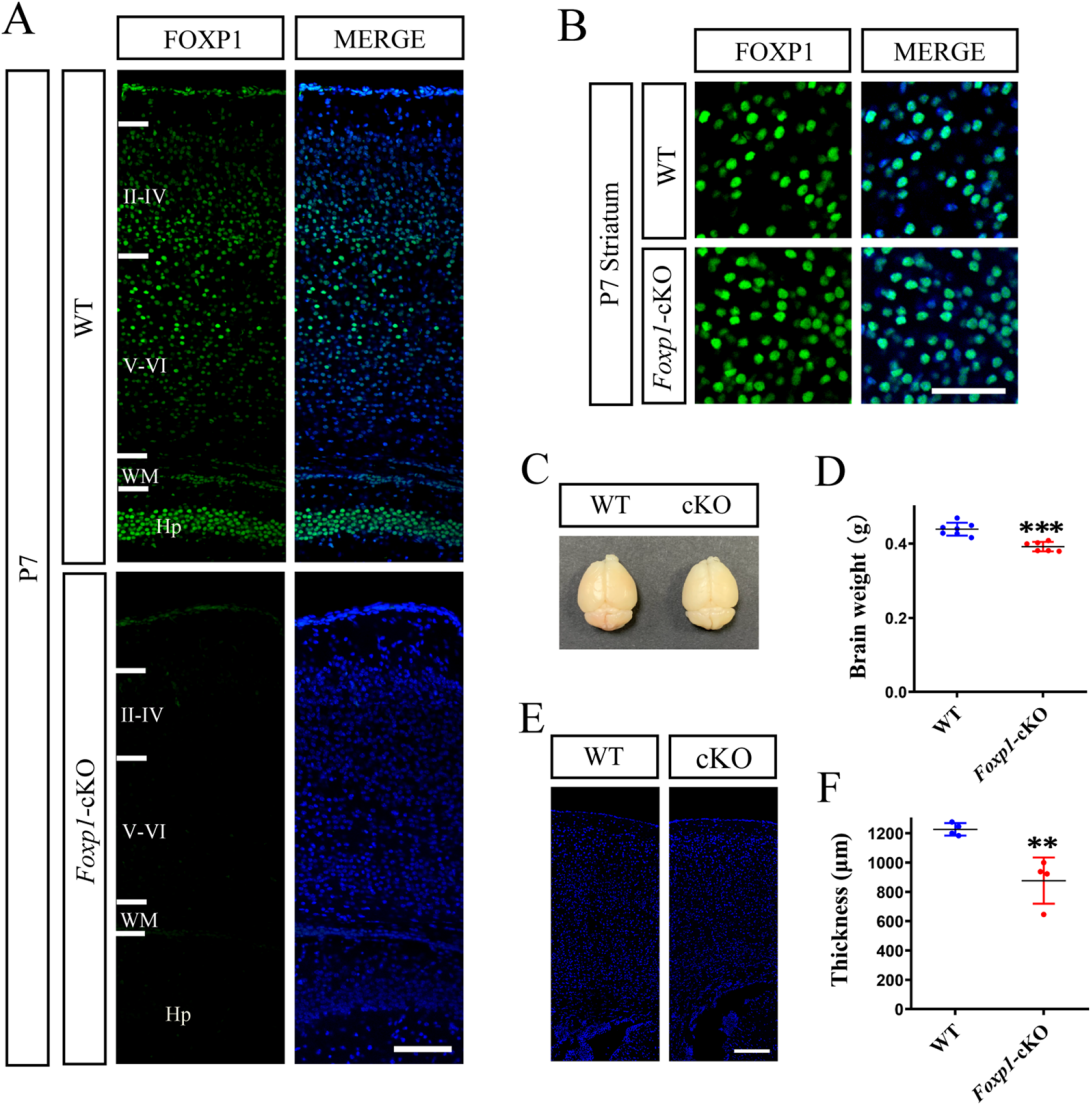
Cortical knockout of *Foxp1* impairs development of cerebral cortex. **A** Immunostaining of FOXP1 (green) on the coronal sections of P7 mouse brains. Nuclei were stained with DAPI (blue). *Foxp1* was absent from the cortex and hippocampus of *Foxp1*-cKO mouse brains. **B** *Foxp1* was expressed in the striatum of both WT and KO mouse brains. II-VI, cortical layers; WM, white matter; Hp, hippocampus. **C** Representative image of whole brains from P35 WT and *Foxp1*-cKO mice. **D** Histogram of brain weights. Circles (Blue: WT; Red: *Foxp1*-cKO) represent the single data points for each brain (n = 6–7 animals). ***p < 0.001. **E** Coronal sections of cerebral cortex from P35 WT and *Foxp1*-cKO mice. Scale bar, 50 μm in (**A**), (**B**), and (**E**). **F** Histogram of the cortical thickness at P35. Circles represent the average thickness for each animal, n = 4 brains per genotype. **p < 0.01

We generated cortex-specific *Foxp1* conditional knockout (*Foxp1*-cKO) mice and control littermates by crossing *Foxp1*^*flox/flox*^ mice with *Emx-Cre* driver as described previously [29, 30]. Under the *Emx1* locus, Cre recombinase is expressed in progenitors and pyramidal neurons in the neocortex and hippocampus [38]. To validate the knockout of *Foxp1* expression, we performed immunofluorescent staining on coronal brain sections (Fig. 1). *Foxp1* was mainly expressed in the cortical plate and hippocampus in wild-type littermates. However, no noticeable signal was detected in these regions of *Foxp1*-cKO mice (Fig. 1A). *Foxp1* was present in the striatum of both WT and KO mouse brains (Fig. 1B), confirming that the *Foxp1* was specifically inactivated in the dorsal telencephalon. *Foxp1*-cKO mice were viable and displayed a grossly normal appearance as WT littermates. However, the KO brain weighed less than WT at postnatal day 35 (Fig. 1C, D). Consistently, the cortical plate appeared thinner than the WT (Fig. 1E, F).

**Fig. 2:**
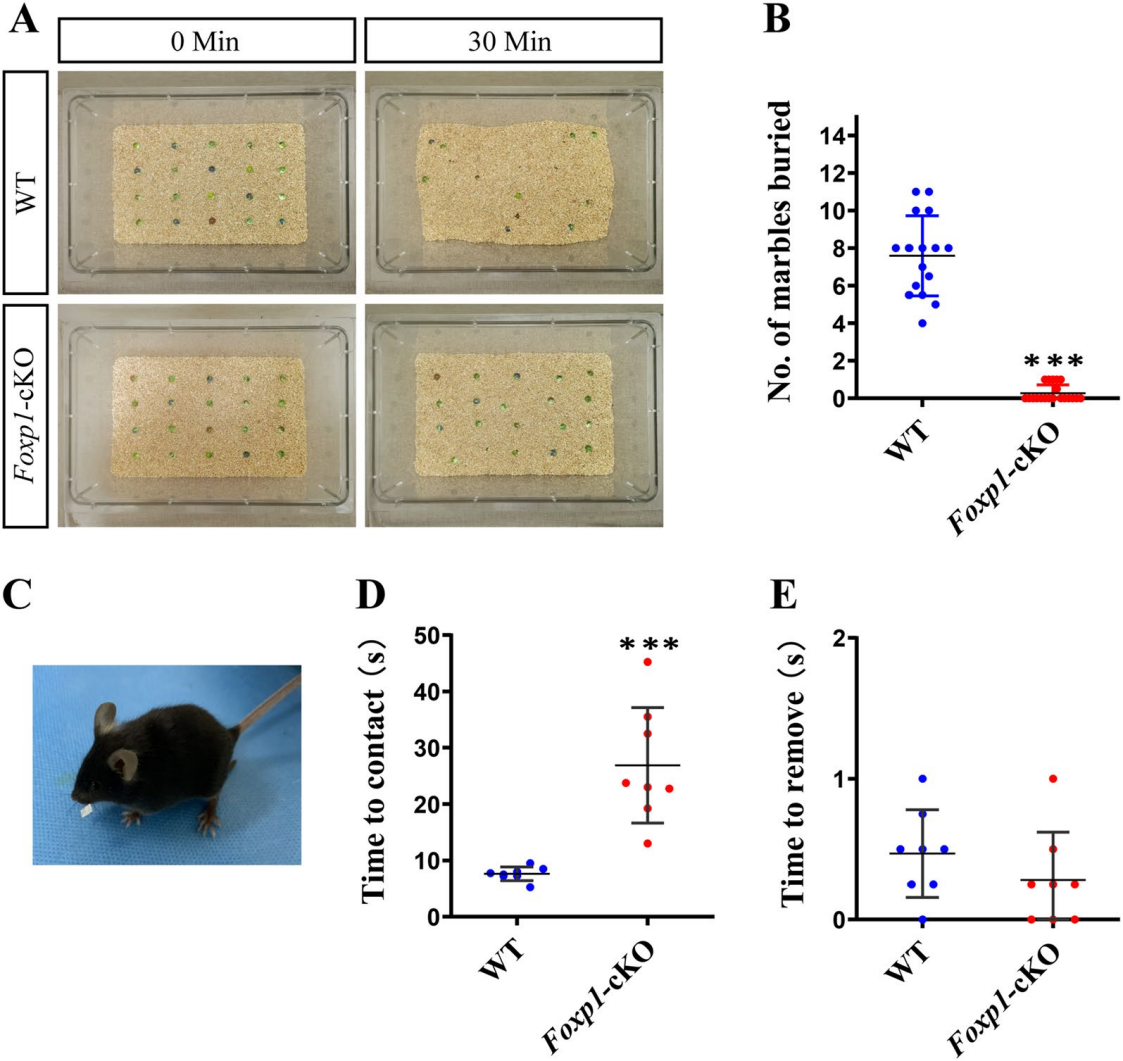
*Foxp1*-cKO mice display tactile sensory deficits. **A** Representative images showing the marbles on top of bedding initially and after 30 min of the marble burying test. **B** Comparisons of the number of buried marbles by the mice. Circles represent the data points from each animal, WT: n = 16; cKO: n = 20. ***p < 0.001. **C** Representative image depicting a mouse undergoing the adhesive removal test. **D** and **E** is the time to contact (**D**) and time to remove (**E**) the adhesives, respectively, in WT (n = 8) and *Foxp1*-cKO mice (n = 8). Circles represent the average time of four trials from each animal. ***p < 0.001

In an open field test, *Foxp1*-cKO mice traveled more distance but spent less time in the field center than the WT littermate controls [29]. The data demonstrated that *Foxp1*-cKO mice had increased locomotor activity and showed more anxiety. To investigate the relevance of cortical *Foxp1* regarding other ASD-associated phenotypes, we evaluated *Foxp1*-cKO mice by the marble burying test [39]. WT mice placed in a cage with marbles on the top of fresh bedding tended to dig and bury the marbles under the bedding. However, *Foxp1* deficiency mice showed much less digging and nudging activity, thereby burying significantly fewer marbles than the WT (WT, 7.59 vs cKO, 0.23, Fig. 2A, B). Marble burying assesses repetitive digging behavior and depends on an animal’s interest in the external environment [39, 40]. To determine if the cortical expression of *Foxp1* is essential for sensory processing, we put the mice on the adhesive removal test, which measures sensorimotor impairments in rodents. When a small adhesive paper was placed on the right or left vibrissae of the mouse (Fig. 2C), the WT animal raised its forelimbs in less than 10 s (time to contact) and swiped off the paper almost immediately (time to remove). However, *Foxp1*-cKO mice needed a much longer time to contact (26.88 s) than the WT (Fig. 2D), indicating that *Foxp1*-cKO mice displayed a lag in response to the presence of adhesive tape. There was no significant difference in the time to remove between WT and cKO mice (Fig. 2E), consistent with the suggestion that KO of *Foxp1* did not cause motor impairment [29]. Thus, the results support that the KO mice may have a tactile sensory deficit.

### *Foxp1*-cKO mice display sensory hyper-responsiveness

**Fig. 3:**
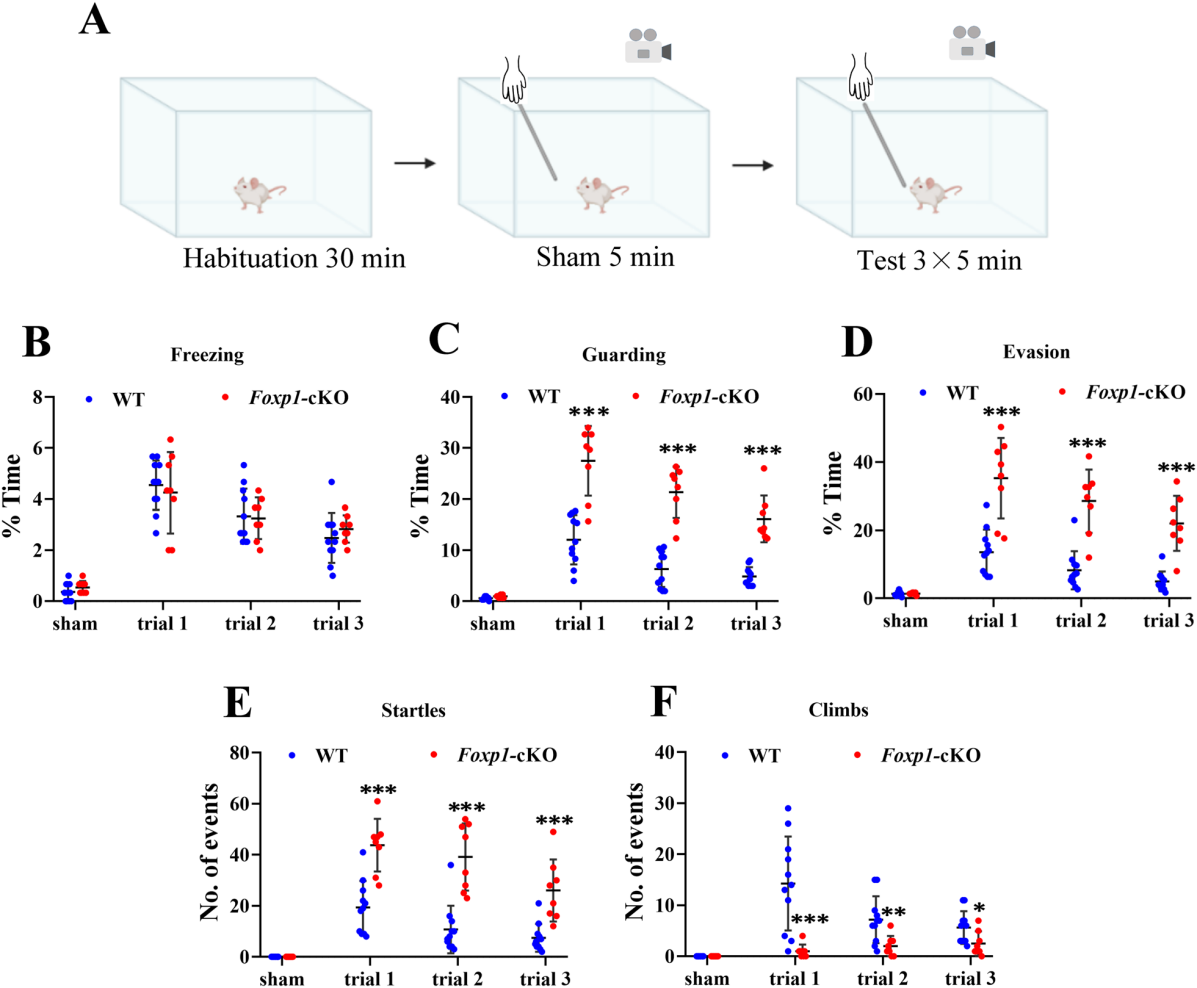
*Foxp1*-cKO mice display hyper-reactive to repeated whisker stimulation. **A** Schematic diagram of whisker nuisance task. The mouse behaviors in response to the whisker stimulation were scored using criteria described in the methods. **B**–**D** Quantification of the average time spent in freezing, guarding, and evasion behaviors of WT (n = 11) and *Foxp1*-cKO mice (n = 8). Circles represent the data points from each animal. ***p < 0.001. **E**–**F** Quantify the number of startle and climb events in response to stick during each session. Circles represent the data points from each animal. *p < 0.05, **p < 0.01, ***p < 0.001

To further investigate the effect of *Foxp1* KO on tactile responses, we compared WT and *Foxp1*-cKO mice by whisker nuisance (WN) test. After 5 min sham-stimulation, the mouse’s whiskers were continuously deflected using a wooden stick for three consecutive sessions (5 min/session) (Fig. 3A). To quantify the behavioral responses to stick presentation, five different behavior responses (freezing, guarding, evasion behaviors, climbing, and startles events) were analyzed. WT and *Foxp1*-cKO mice had comparable responses when the stick approached the animal’s head but avoided contact with whiskers (sham stimulation). From the first to third trials of the whisker stimulation, *Foxp1* cKO mice and the control had a similar level of freezing behavior (Fig. 3B). However, in response to the repeated whisker stimulation, more time *Foxp1*-cKO mice showed guarding (trial 1, 27.50%; trial 2, 21.33%; trial 3, 16.12%) and evasion behaviors (trial 1, 35.29%; trial 2, 28.54%; trial 3, 22.04%) than WT controls did (Guarding: trial 1, 12.06%; trial 2, 6.30%; trial 3, 4.85%. Evasion: trial 1, 13.55%; trial 2, 8.24%; 1.50; trial 3, 5.00%) (Fig. 3C, D), indicating that *Foxp1* deficiency mice were easier to get into a defensive state. Moreover, *Foxp1*-cKO mice exhibited more sudden and uncoordinated avoidance movement (startling), indicating they were frightened by the wooden stick. On the other hand, *Foxp1*-cKO mice showed fewer climbing events than WT controls (Fig. 3F), supporting that the mice may have a reduced interest in the environment (stick) [35]. The data demonstrate that *Foxp1* deficiency leads to tactile hyper-reactive or a fight-or-flight response to the whisker stimulation, particularly in the first session. However, both WT and *Foxp1*-cKO mice had a gradual reduction of the scores from the first to the third trial, indicating that the KO did not change the habituation process to the repetitive stimulation.

### *Foxp1 *KO induces fewer *c-Fos* immunoreactivity in cortical layer IV but more in basolateral amygdala

**Fig. 4:**
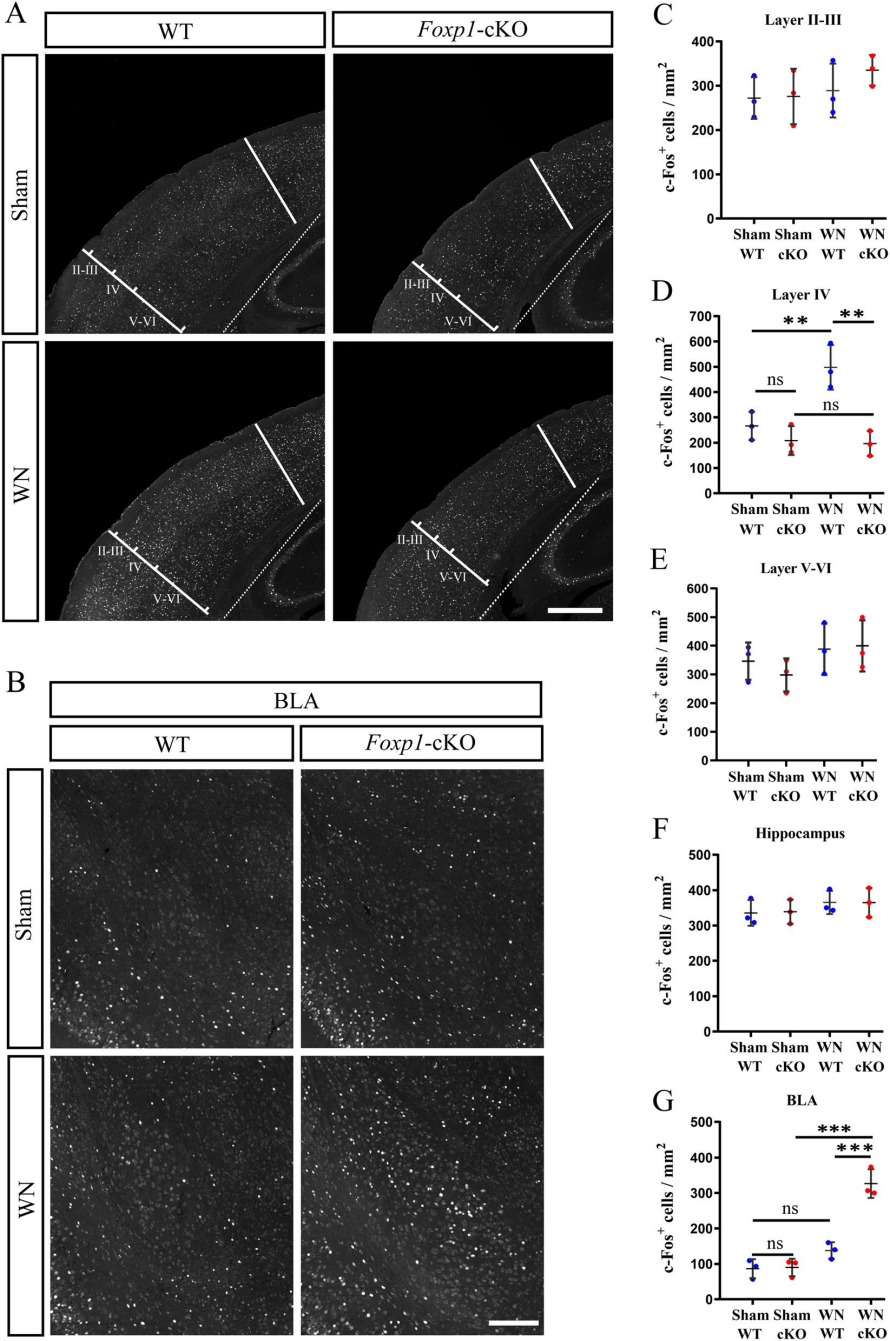
C*-*Fos is not induced in layer IV but increased in BLA of *Foxp1*-cKO mice by whisker stimulation. **A**–**B** c-Fos immunolabeling in the barrel cortex (**A**), hippocampus (**A**), and BLA (**B**) of WT and cKO mice 2 h following repeated whisker stimulation. White dot lines indicate the border between the cortex and hippocampus. Scale bars, 500 μm in (**A**), 200 μm in (**B**). **C**–**E** Quantification of c-Fos positive cells in cortical layers of WT and *Foxp1*-cKO mice. Circles (Blue: WT; Red: *Foxp1*-cKO) represent the average cell number for each animal (3 animals per genotype; 3 sections per animal). Ns, no significant difference; **p < 0.01. **F**–**G** Statistics of c-Fos positive cells in the hippocampus (**F**) and BLA (**G**). Circles represent the c-Fos^+^ cell density for each animal (3 animals per genotype; 3 sections per animal). Ns, no significant difference; ***p < 0.001

Repeated whisker stimulation may induce activation of immediate early gene *c-Fos* expression in layer IV neurons of S1 [19, 41]. We investigated activity-dependent c-Fos immunoreactivity two hours after the WN test was completed. The c-Fos expression following the sham session (basal expression) was not significantly different across all the regions examined between *Foxp1* KO and control mice. Repeated whisker stimulation upregulated c*-*Fos in layer IV neurons of S1 in WT mice (Sham, 266.00 vs WN, 497.78, Fig. 4D) but not in *Foxp1* KO mice (Sham, 208.33 vs WN, 196.56, Fig. 4D). This finding indicates that *Foxp1* KO diminished neuronal activation in layer IV of S1, a region important for whisker-dependent somatosensory signal processing. *Foxp1* KO and control mice exhibited comparable c-Fos expression in other cortical layers of S1 and the hippocampus before or after the whisker stimulation (Fig. 4C and E, A and F). In contrast, in response to the whisker stimulation c-Fos positive cells were increased in the basolateral amygdala (BLA) of *Foxp1*-cKO mice (Sham, 89.78 vs WN, 326.67) while remaining statistically unchanged in WT (Sham, 86.33 vs WN, 137.78, Fig. 4G). C-Fos activation in BLA was also observed in the autistic model of *En2*^*−/−*^ mice following the WN test [19]. Amygdala is the integrative center for emotional behavior and plays a pivotal role in innate fear. Therefore, the increased neural activity in BLA is consistent with the hyperreactive response to the repeated whicker stimulation in *Foxp1*-KO and *En2*^*−/−*^ mice.

### Loss of *Foxp1* in the cortex disrupts barrel formation in S1

**Fig. 5:**
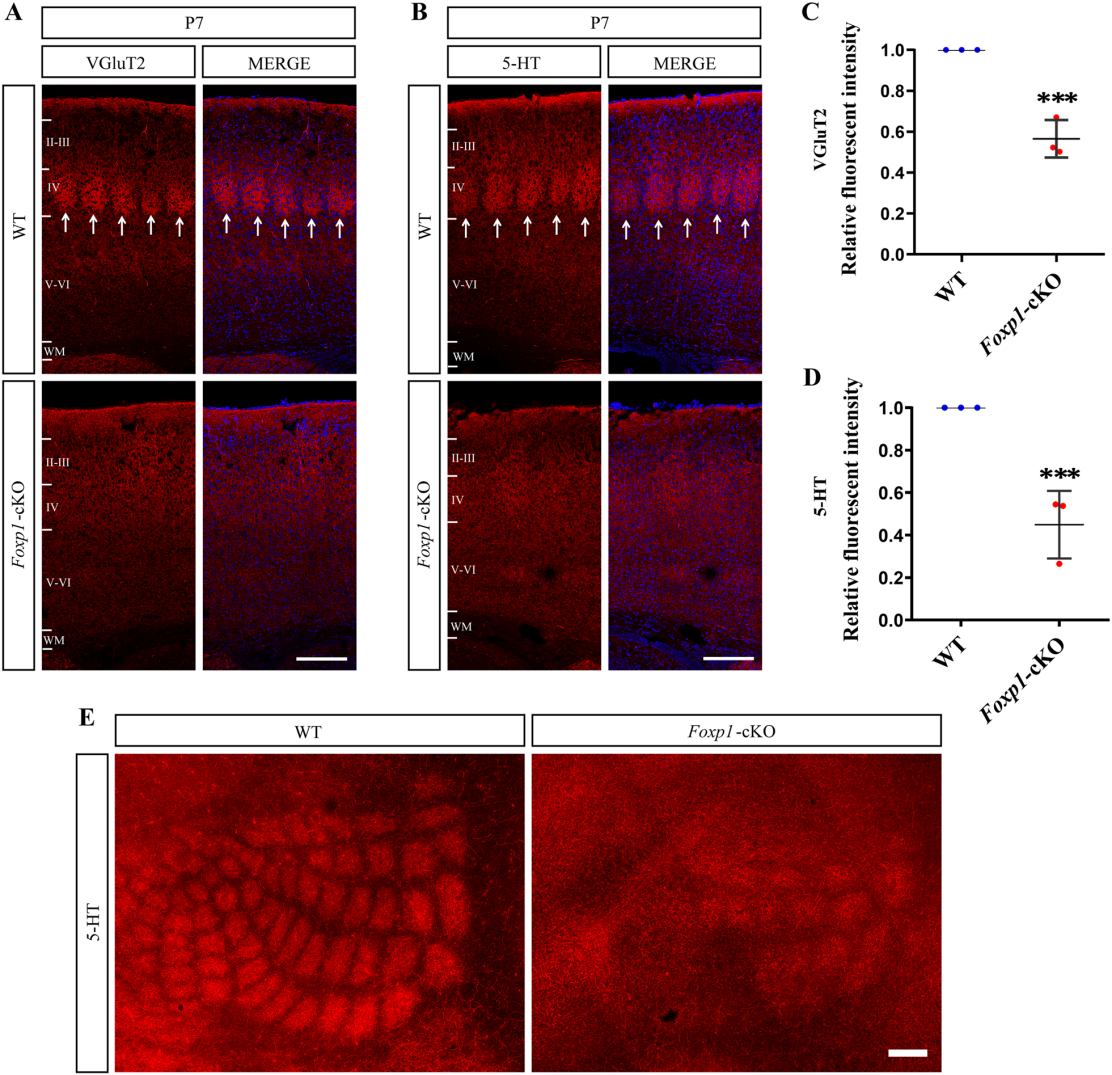
*Foxp1* regulates barrel formation in S1. **A** and **B** Brain coronal sections from P7 WT and *Foxp1*-cKO mice subjected to VGluT2 (**A**) or 5-HT (**B**) immunostaining. Arrow delineates the barrel units in layer IV of S1. The barrel patches from TCA were almost invisible in *Foxp1*-cKO mice. Scale bar, 200 μm in (**A**) and (**B**). **C** and **D** Quantifications of VGluT2 (**A**) and 5-HT (**B**) fluorescence intensity of layer IV. Circles represent the average fluorescence intensity for each animal (3 animals per genotype; 3 sections per animal). ***p < 0.001. **E** A representative image showing 5-HT staining on flattened cortices from P7 WT and *Foxp1*-cKO mice. Scale bar, 200 μm

**Fig. 6:**
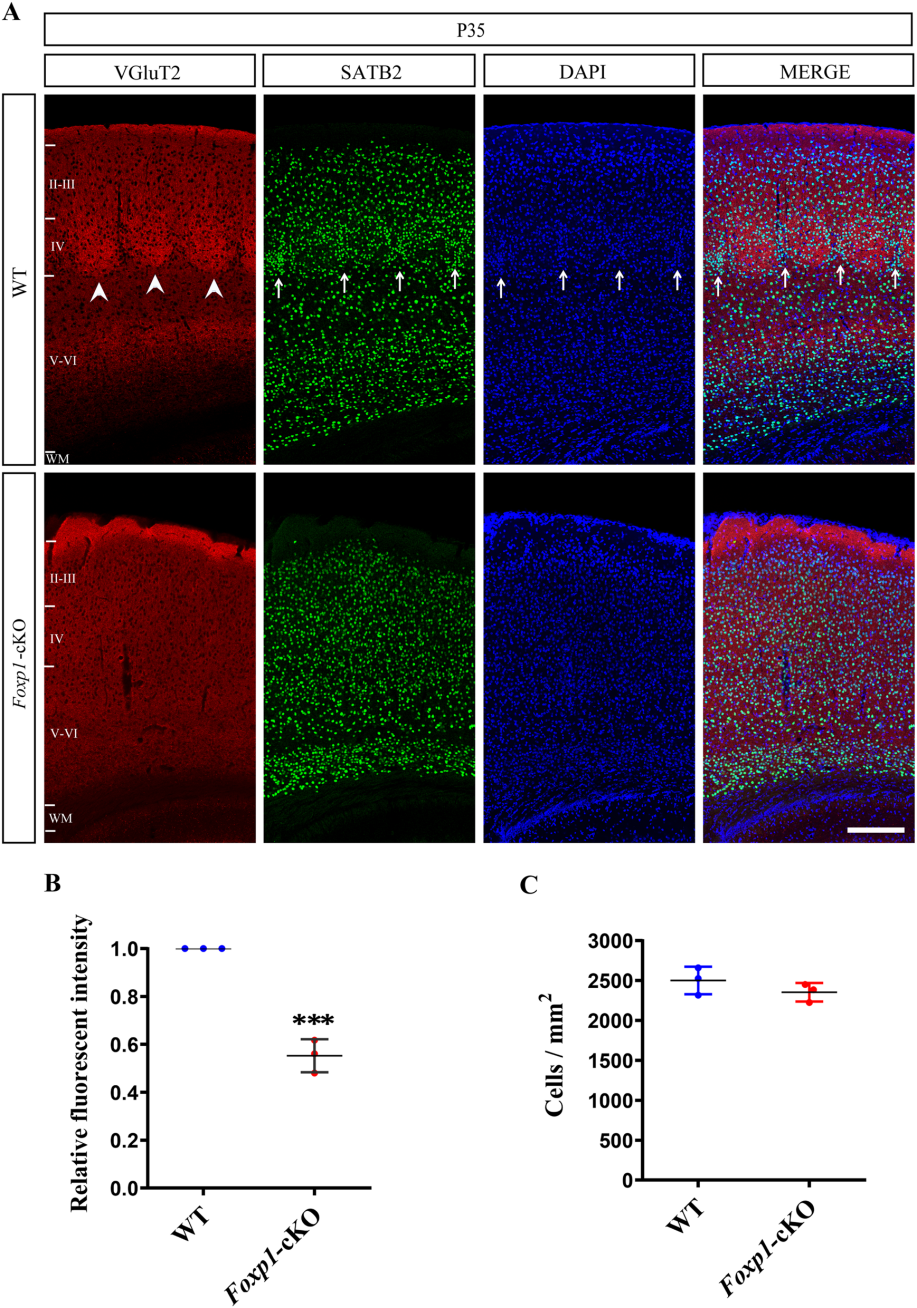
Cortical deletion of *Foxp1* alters cytoarchitecture of barrel field. **A** Double immunolabeling of VGluT2^+^ TCA terminals and SATB2^+^ cortical neurons from P35 WT and the KO mouse brains. DAPI (blue) and SATB2 (red) positive cells formed a ring-like organization around VGluT2^+^ TCA (red) in control mice (Arrow) but not in the *Foxp1*-cKO cortex. Arrowhead delineates the barrel in layer IV of S1. Scale bar, 200 μm. **B** Quantifications of VGluT2 fluorescence intensity of layer IV. Circles represent the average fluorescence intensity for each animal (3 animals per genotype; 3 sections per animal). ***p < 0.001. **C** The density of SATB2^+^ cells in layer IV. Circles represent the average cell density for each animal (3 animals per genotype; 3 sections per animal)

The deficit in c*-*Fos activation in S1 indicates possible disorders of somatosensory connectivity. Since *Foxp1* is expressed in layer IV of S1 [42], we set out to determine whether the cortical KO of *Foxp1* disrupts the formation of the sensory map. A barrel-like structure appears as early as P5 when thalamocortical afferents segregate and respond preferentially to individual whiskers [43]. We performed immunostaining of VGluT2, a vesicular glutamate transporter specifically expressed in TCA terminals, on coronal sections from the P7 brain. In WT brains, VGluT2 staining exhibited discrete, patch-like clusters in layer IV of the barrel cortex. In contrast, TCA was distributed diffusively in the KO mice. Barrel patterns or patch structures were almost missing in *Foxp1*-cKO brains (Fig. 5A). In addition, VGluT2 signal intensity was significantly lower in layer IV of S1 in *Foxp1*-cKO mice compared to control littermates (Fig. 5C). To confirm the patchless phenotype, we further identified the barrel structures by immunostaining with serotonin antibody, which labels presynaptic terminals from TCA in the early postnatal cortex. Similarly, control mice displayed barrel-like structures that disappeared in the *Foxp1*-cKO brains. The 5-HT staining intensity in the *Foxp1*-cKO brain was only half of the WT. (Fig. 5B and D). We also visualized the TCA clustering on flattened cortices by 5-HT staining. *Foxp1* KO brains, but not the WT, lost the organized patches in S1 (Fig. 5E). Finally, the TCA organization was examined by VGluT2 staining in P35 brains. No barrel structures were detected in the S1 of *Foxp1*-cKO mice, in contrast to the WT (Fig. 6A), suggesting that the patchless was not caused by a delayed arrival of TCA in layer IV. These data demonstrate that cortex-specific deletion of *Foxp1* is sufficient to disrupt the whisker-related barrel pattern or the somatosensory map.

To examine cytoarchitectural disruption brought by the KO, we immunostained the S1 by nuclear marker DAPI and upper-layer marker SATB2, in combination with VGluT2. In the control brain, layer IV neurons accumulated along the barrel walls, forming a ring-like distribution around the thalamocortical afferents. The density of layer IV cells on the boundary of the barrel was higher than the hollow circle within. However, when *Foxp1* was deleted in the cortex, layer IV neurons distributed roughly even across the cortical sections (Fig. 6A). Quantification analysis showed that the loss of *Foxp1* altered the distribution but not the average density of the neurons in layer IV (Fig. 6C), consistent with a previous observation that loss of *Foxp1* does not affect the thickness and number of the upper layer cells [44]. The data supported that the loss of *Foxp1* disrupted barrel structures and, as a result, the thalamocortical connectivity, leading to diminished c-Fos activation in layer IV (Fig. 4).

### *Foxp1* is essential for dendritic spines and synaptic connections

**Fig. 7:**
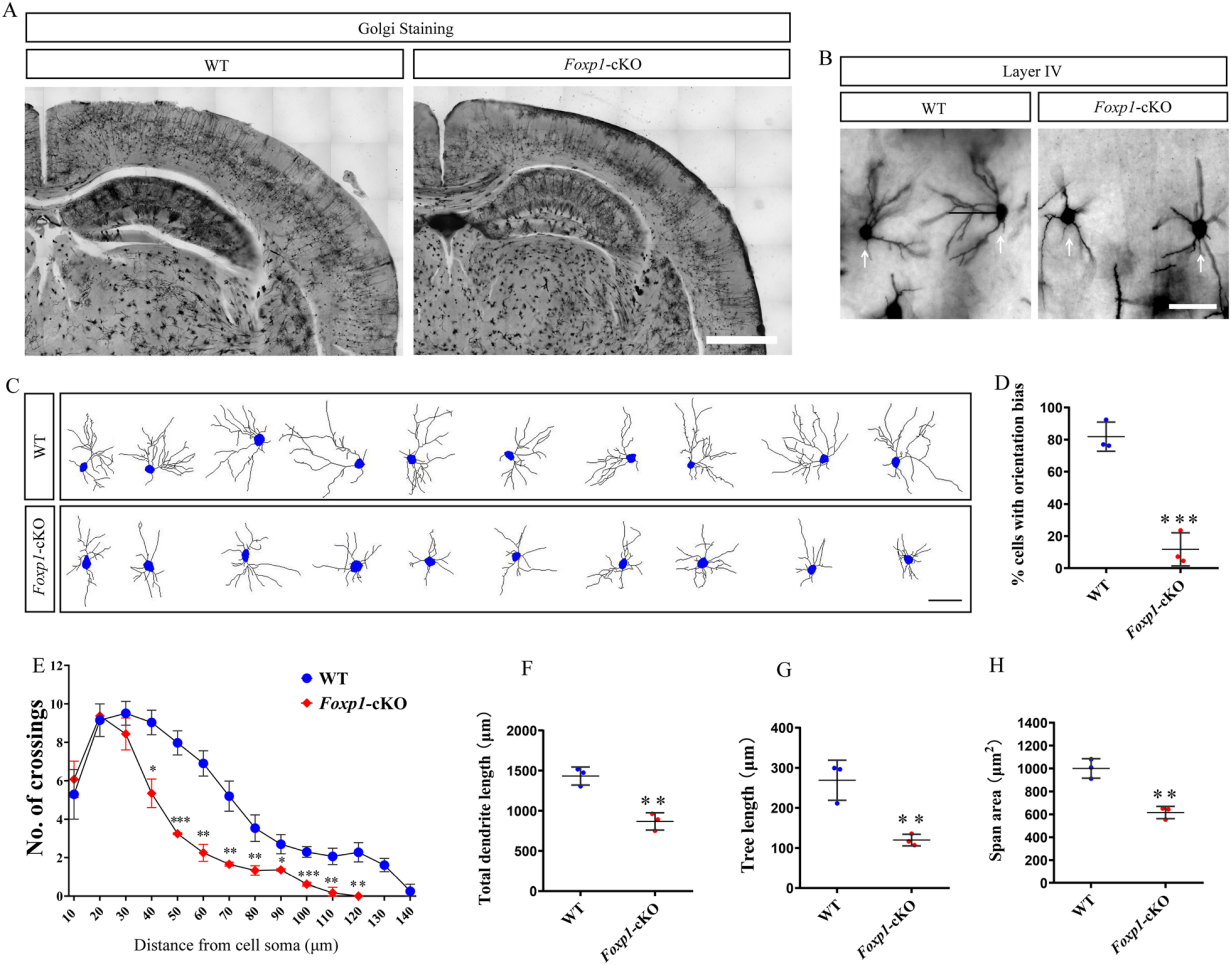
*Foxp1* is required for the dendrite development of layer IV neurons. **A** Representative Golgi-Cox staining in *Foxp1*-cKO and WT control mice at 6 weeks of age. Scale bar, 1 mm. **B** Photomicrographs of spiny stellate neurons (arrows) stained with Golgi-Cox in the barrel cortex. Scale bar, 50 μm. **C** Reconstruction of individual layer IV stellate neurons from WT and the KO brains. Scale bar, 50 μm. **D** Histogram shows the proportions of cells with asymmetric dendrite orientation. Symbols (Blue circles: WT; Red circles: *Foxp1*-cKO) represent the single data points for each animal (13–25 cells per animal, n = 3 animals per genotype). ***p < 0.001. **E** The number of dendritic intersections with Sholl circles at increasing distances from the center of the cell soma. The KO mice had significantly fewer intersections with circles 40 to 120 μm away from the soma. Circles represent the average of all analyzed cells for each animal (13–25 cells per animal), n = 3 brains per genotype. *p < 0.05, **p < 0.01, ***p < 0.001. **F**–**H** Quantification of the total length of dendrites, span area, and tree length of layer IV neurons in S1. Circles (Blue: WT; Red: *Foxp1*-cKO) represent the average of all analyzed cells for each animal (13–25 cells per animal), n = 3 brains per genotype. **p < 0.01

When S1 neurons migrate to layer IV, the neurons reorganize around the incoming TCA, and the dendrites form synaptic connections with TCA terminals. The cortical neurons display an asymmetric branching morphology with the dendrites orientating toward TCA [45]. To evaluate the distribution of dendrites and spines, we employed Golgi staining to analyze the spiny dendrites from excitatory stellate neurons in layer IV (Fig. 7A). The morphology of neurons was individually imaged and reconstructed (Fig. 7B, C). *Foxp1* KO mice were clearly distinguished from the WT in their dendritic orientation. In control mouse brains, 81.81% of the cells exhibited asymmetric branching with dendrites projecting toward the barrel center, while only 11.74% of the cells displayed such orientation bias in *Foxp1*-cKO mice (Fig. 7D). We also studied the complexity of dendrites by Sholl analysis. Compared to the control, *Foxp1*-cKO neurons had fewer intersections between dendrites and the Sholl circles located 40 to 140 μm away from the soma center (Fig. 7E). Moreover, the total dendritic length, dendritic surface area, and the tree length of layer IV spiny neurons were decreased in *Foxp1*-cKO mice compared to WT (Fig. 7F, H). These data demonstrate that cortex-specific deletion of *Foxp1* perturbed the dendrite orientation and outgrowth of the layer IV neurons.

**Fig. 8:**
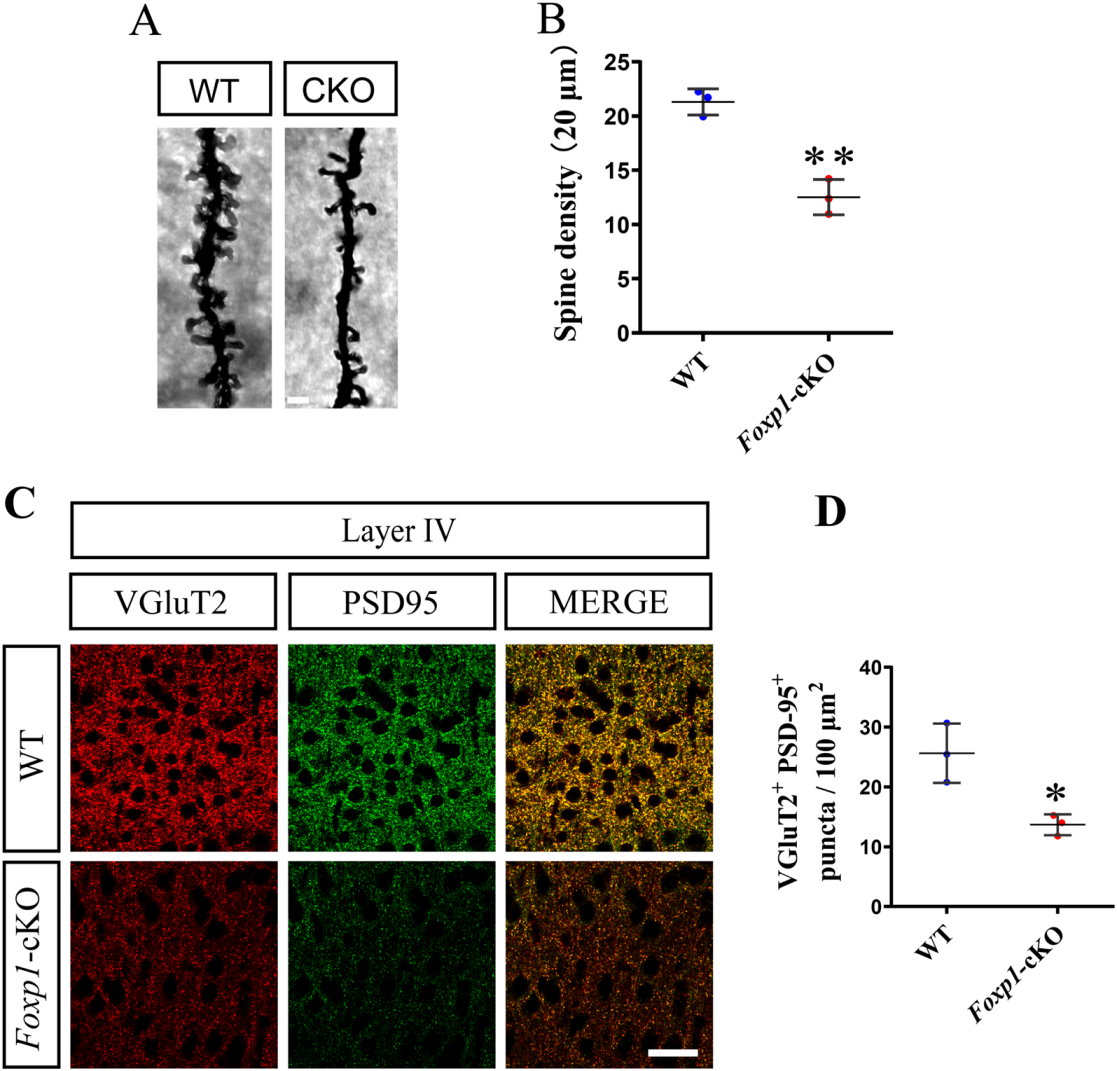
*Foxp1*-cKO mice display fewer thalamocortical synapses. **A** Representative images of spines on dendritic branches of the spiny stellate neurons from KO and WT mice. Scale bar, 2 μm. **B** Histogram of the mean spine counts on 20 μm long dendrites. Circles (Blue: WT; Red: *Foxp1*-cKO) represent the average density of all analyzed segments from each animal (12 segments per animal), n = 3 brains per genotype. **p < 0.01. **C** Brain coronal sections were immunostained for VGluT2 and PSD-95. Scale bar, 20 μm. **D** Statistics of puncta number of VGluT2^+^PSD-95^+^ excitatory thalamocortical synapses per 100 mm^2^ in layer IV of S1. Circles represent the average puncta number for each animal (3 animals per genotype; 3 sections per animal). *p < 0.05

Moreover, the spine density from randomly selected dendrite segments in the *Foxp1*-cKO mice was lower than that of the control (WT, 21.30 vs cKO, 12.52, Fig. 8A, B), indicating that the transcription factor may regulate spinogenesis or stability of the spines. The loss of postsynaptic spines should have a detrimental effect on the synaptic connection. We thus examined excitatory thalamocortical synapses by co-immunostaining VGluT2 with PSD-95, a postsynaptic protein. In agreement with the decreased density of spines, the VGluT2^+^ PSD-95^+^ puncta in S1 were reduced in the *Foxp1*-cKO mice compared to WT (WT, 25.44 vs cKO, 14.00, Fig. 8C, D). *Foxp1* deficiency may impede the growth or maintenance of dendritic spines in cortical neurons, leading to fewer thalamocortical synapses in S1.

## Discussion

Atypical sensory behaviors are a common feature of ASD [46, 47]. This study found that loss of the autism-associated gene *Foxp1* in the mouse cortex leads to a slower reaction to adhesive paper and hyper-responsiveness to repeated whisker stimulation (Figs. 2 and 3). The aberrant responses were accompanied by diminished c-Fos activation in the input layer of S1 and increased c-Fos in BLA (Fig. 4). We postulate that the *Foxp1*-cKO mice may have tactile sensory deficits (hyposensitivity). The hyper-reactivity evoked by the repeated stimulation may reflect fearful and emotional responses directly controlled by BLA, a core structure in the limbic system. Changes in sensory processing have been observed in other mouse models of ASD. *Fmr1* KO mice similarly exhibit hyper-reaction in response to the whisker stimulations [14]. *En2*^*−/−*^ mice also display somatosensory hyper-responsiveness as assayed by the WN test [19]. Unlike previous studies of *En*2 and *Fmr1* performed on traditional knockout mice, this study on *Foxp1* pinpointed the cerebral cortex as an origin of atypical sensory behavior in mice lacking ASD-related genes.

Tactile sensory abnormality in *Foxp1*-cKO mice was accompanied by defective barrel formation that is essential for thalamocortical connectivity. Barrel cortex development requires the coordinated maturation of presynaptic TCA and postsynaptic layer IV cortical neurons. Activity-dependent glutamatergic and serotonergic neurotransmission is instructive for thalamocortical innervations and barrel patterning [48, 49]. On the other hand, intrinsic transcription factors Bhlhe22/Bhlhb5 [50], Eomes [51, 52], Ctip1 [53], and Satb2 [54] have been implicated in early barrel development. We found that FOXP1 deficiency in the cerebral cortex perturbs the dendrites and spines of layer IV neurons, leading to diminished synapses and barrel formation (Figs. 5, 6, 7 and 8). Consistently, whisker stimulation cannot fully activate the expression of c-Fos in the major input layer of cortex (Fig. 4). This study presented anatomical data showing reduced thalamocortical connectivity and defective barrel formation in a genetic mouse model of ASD.

The molecular mechanism underlying the *Foxp1* regulation of dendrites and spines in spiny stellate neurons may be multifactorial. *Foxp1* in the forebrain controls multiple downstream targets, including *CNTNAP2*, *CTTNBP2,* and *RORβ* [44]. CNTNAP2, a protein associated with autism, helps to stabilize newly formed dendritic spines in the forebrain [55]. CTTNBP2 modulates dendritic arborization by adjusting F-actin organization and microtubule stability [56]. Knockdown of *Cttnbp2* in cultured neurons decreases the spine density and causes ASD-like behaviors in mice [57–59]. *RORβ* is expressed highly in layer IV of the central nervous system, notably S1 [60]. RORβ protein expression is correlated with barrel formation. Neuronal clusters induced by *RORβ* overexpression are specifically innervated by thalamocortical fibers [61]. Thus, *Foxp1* may be a central regulator of molecules involved in the spine and barrel formation.

Sensory hyper-reactivity in ASD patients is associated with alterations in structural and functional brain connectivity [62]. The relationship between the loss of thalamocortical connection and hyper-reactivity has not been established. Studies from *Fmr1* KO mice by in vivo two-photon calcium imaging found that close to half of the neurons in layer 2/3 of S1 lost their adaptation to repetitive whisker stimulation, which may contribute to somatosensory hyper-responsiveness in autism [62]. However, *Foxp1*-cKO mice in this study exhibited a comparable gradual reduction of the WN test scores as the WT, suggesting that the mice can habituate to repetitive stimulation of whiskers (Fig. 3). Although multiple pathways may exist, a change in the thalamo-cortical-amygdala circuit might lead to hyper-reactivity as postulated below.

*Foxp1* is expressed in excitatory projection neurons but not in the interneurons of the cerebral cortex [63]. The number of excitatory thalamocortical synapses in layer IV was significantly decreased in *Foxp1*-cKO mice compared with control mice (Fig. 8). Compromised excitatory neural input may alter excitatory/inhibitory (E/I) balance in the cerebral cortex. E/I imbalance has been implicated in the etiology of autism [64]; for example, reduced excitatory synaptic transmission in pyramidal neurons of mouse prefrontal cortex gives rise to the social and vocalization deficits in ASD [64]. The cerebral cortex is essential for suppressing innate defensive behavior [65]. A decrease in excitatory or an increase in inhibitory neural activity in the cerebral cortex, specifically the prefrontal cortex, would lead to disinhibition of the amygdala. This is supported by the increased activation of BLA (Fig. 4B and G). Amygdala receives sensory information directly from the thalamus before reaching the cortex, and is a core neural structure for processing fearful and threatening stimuli [66, 67]. Activation of the amygdala may lead to a fight-or-flight response in the animal.

### Limitations

This study presented robust evidence for disruption of the barrel cortex by cortical deletion of an ASD-related gene. However, we only examined limited methods to trigger tactile sensory responses, and *Foxp1*-cKO mice overreacted to the repeated whisker stimulation. We do not know if the mice may react differently to other forms of tactile stimulation. In addition, the relationship between BLA and hyper-reactivity is associative, and whether it is causative remains to be determined. It is unclear how the defective thalamocortical connection gives rise to the hyper-reactive response. Future experiments with electrophysiological recording are needed to analyze the function of thalamo-cortical-amygdala circuits in the disinhibiting amygdala and enhancing fearful responses in animal models of autism.

## Conclusions

Overall, our findings show that the cortical deficiency of *Foxp1* leads to defective barrel formation. The loss of barrel formation is a reminiscence of the brain structural changes from maternal separation [68], a condition leading to developmental delay and ASD in human and animal models [69, 70]. Thus, genetic changes and early adversity may have a shared role in altering thalamocortical connectivity. *Foxp1*-cKO mice have tactile sensory deficits while exhibit hyper-reactivity and avoidance behavior, which would exaggerate social and communication difficulties in ASD. This study presents anatomical evidence for reduced thalamocortical connectivity in a genetic mouse model of ASD and demonstrates that the cerebral cortex can be the origin of atypical sensory behaviors.

## Data Availability

All data and data analysis associated with this study are available upon request.
